# Supplementary material for: Adenosine-Specific Transcriptional Programs in Murine Connective Tissue-Type Mast Cells
Source: ACS Pharmacol Transl Sci. 2026 Jan 8;9(2):430–46. doi: 10.1021/acsptsci.5c00741 (PMC12910494; doi:10.1021/acsptsci.5c00741)
Supplement: Supplementary file 1 [file pt5c00741_si_001.pdf]

Supporting information to:

Adenosine-specific transcriptional programs in murine connective tissue type mast cells

Qihua Liang[1,2†], Volodymyr Tsvilovskyy[1,2†], Anouar Belkacemi[1,2], Merima Bukva[1], Christin Richter[1], Nicole Ludwig[3,4], Andreas Keller[3,5,6], Marc Freichel[1,2]**

[1]Institute of Pharmacology, Heidelberg University, 69120 Heidelberg, Germany
[2]DZHK (German Centre for Cardiovascular Research), partner site Heidelberg/Mannheim, 69120 Heidelberg, Germany
[3]Clinical Bioinformatics, Saarland University, 66123 Saarbrücken, Germany
[4]Department of Human Genetics, Saarland University, 66421 Homburg/Saar, Germany
[5]Helmholtz Institute for Pharmaceutical Research Saarland, Helmholtz Center for Infection Research, 66123 Saarbrücken, Germany
[6]PharmaScienceHub, Saarland University, 66123 Saarbrücken, Germany

[†] These authors share first authorship
Correspondence*:
Marc Freichel: marc.freichel@pharma.uni-heidelberg.de
Andreas Keller: andreas.keller@ccb.uni-saarland.de

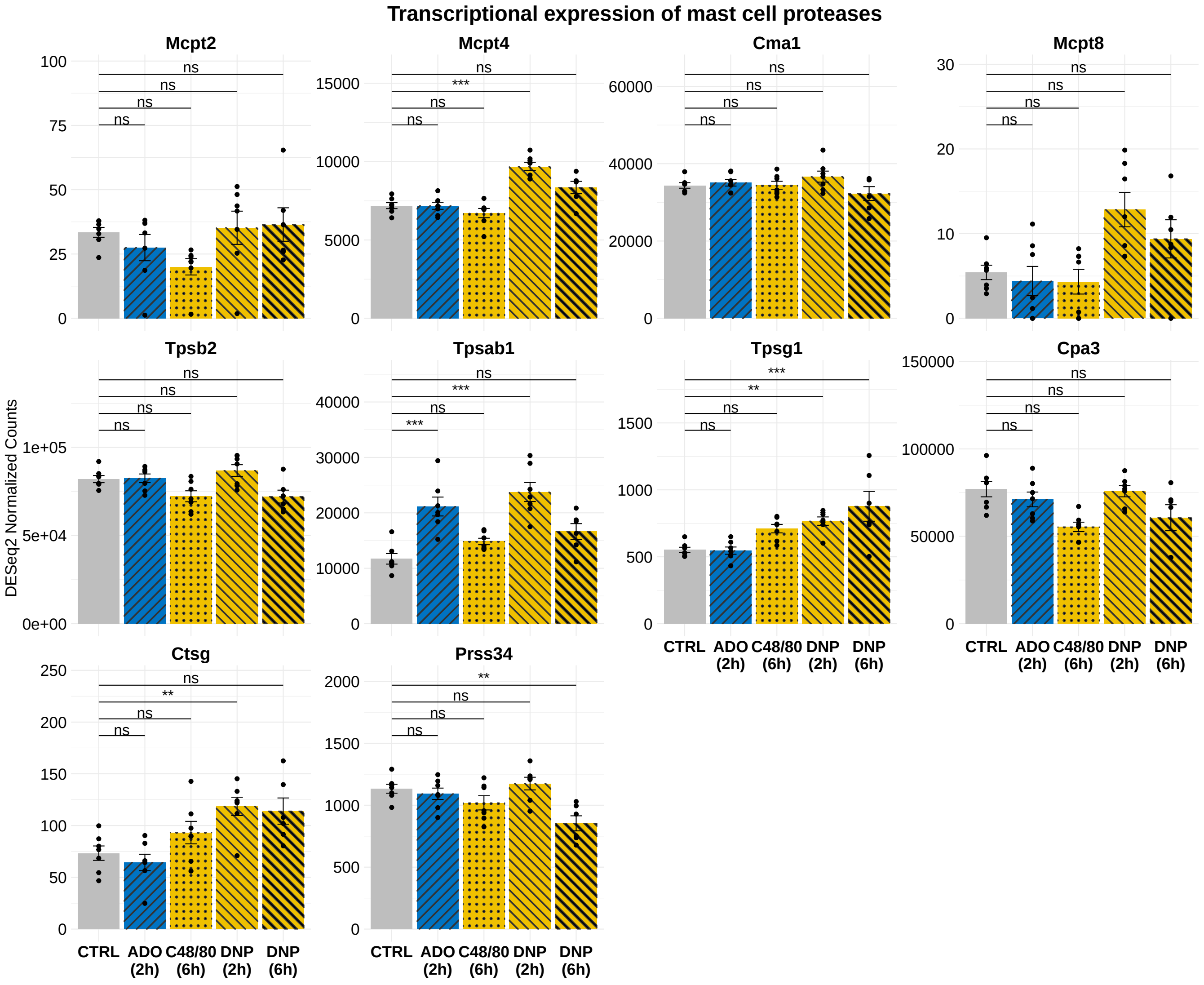

Figure S1: Expression profiling of mast cell proteases in PMCs. Normalized expression plots show mast cell protease gene expression across five conditions: CONTROL (CTRL), ADO (2h), C48/80 (6h), DNP (2h), and DNP (6h). Each condition included seven biological replicates, except for the DNP (6h) treatment, which comprised six replicates due to the unavailability of replicate 2.2. Statistical significance was assessed using DESeq2, with significance levels defined as follows: ns, not significant; $*p_{\text{adj}} < 0.01$; $**\,p_{\text{adj}} < 0.005$; $***p_{\text{adj}} < 0.001$.

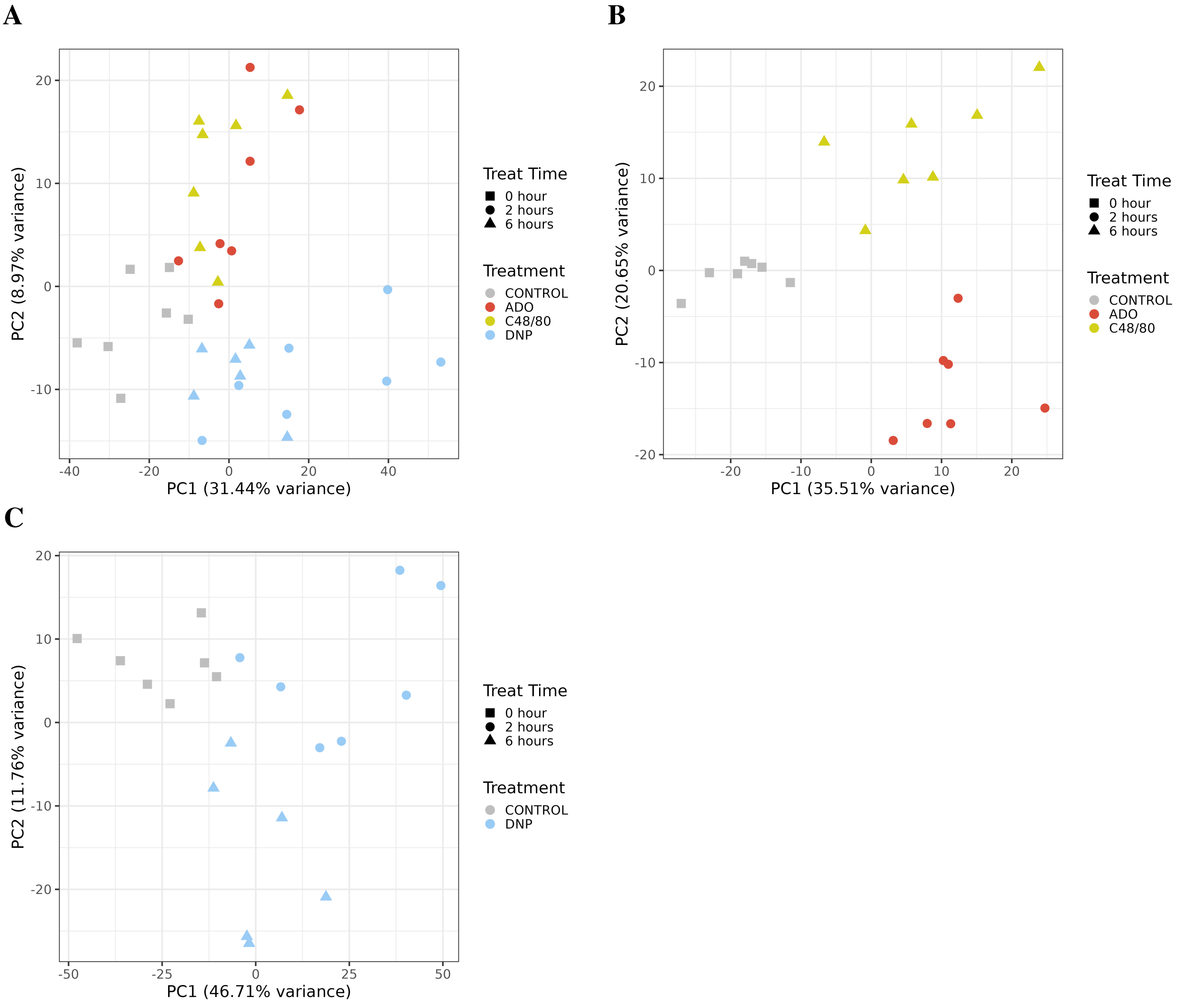

Figure S2: Principal component analysis of transcriptomic profiles following treatment with ADO, C48/80, and DNP. **A** PCA of all five experimental groups: CONTROL, ADO (2h), C48/80 (6h), DNP (2h), and DNP (6h). (B) PCA of CONTROL, ADO (2h), and C48/80 (6h) groups. (C) PCA of CONTROL, DNP (2h), and DNP (6h) groups. Each point represents an individual sample, colored by treatment and shaped according to treatment duration (square = 0 h, circle = 2 h, triangle = 6 h). Percent variance explained by each principal component is indicated on the respective axes.

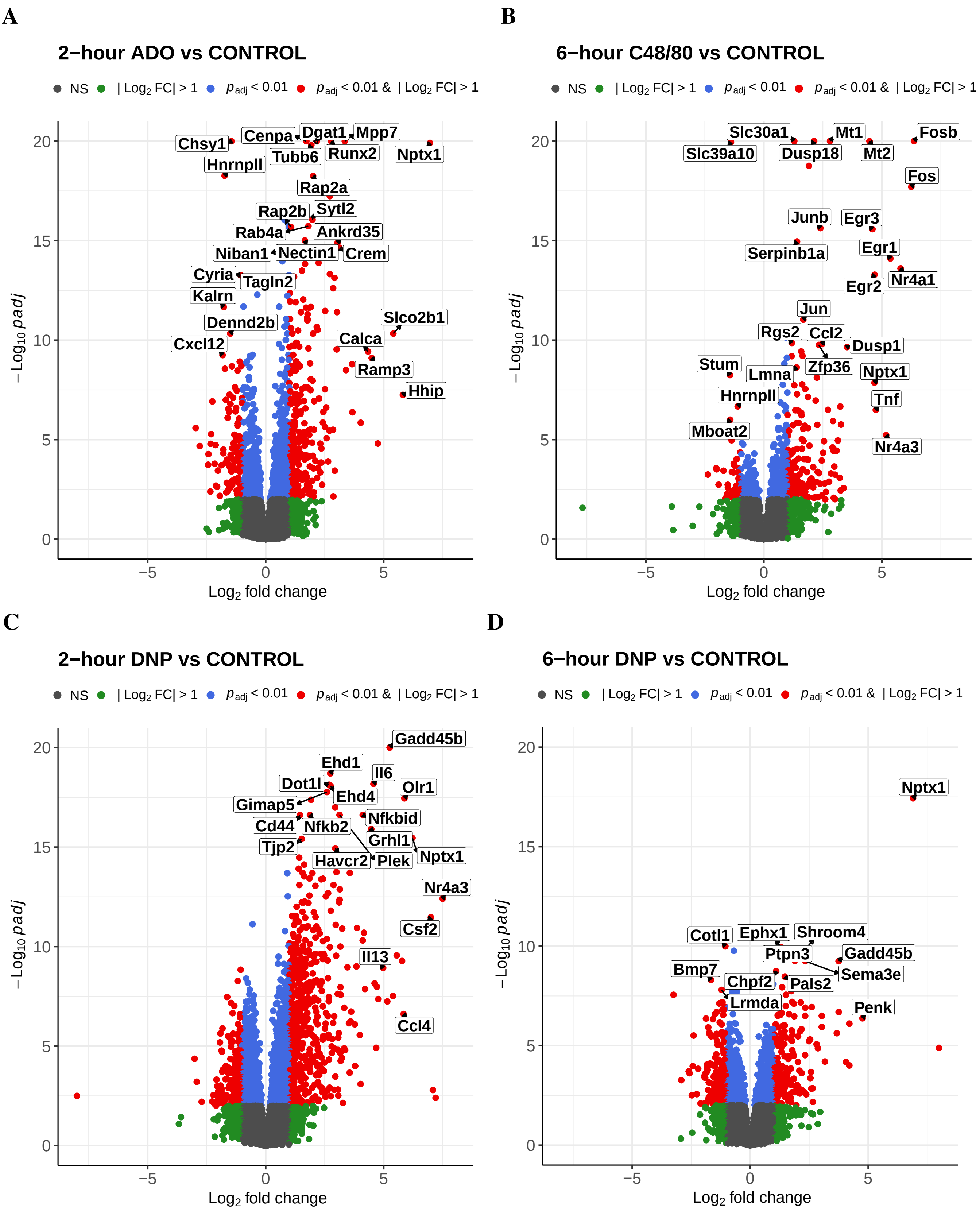

Figure S3: Transcriptomic analysis reveals DEGs in stimulated mast cells
Volcano plot of DEGs in mast cells following **(A)** 2-hour ADO treatment versus CONTROL. **(B)** 6-hour C48/80 treatment versus CONTROL. **(C)** 2-hour DNP treatment versus CONTROL. **(D)** 6-hour DNP treatment versus CONTROL. Significant DEGs fulfills $|\log_2 \mathrm{FC}| > 1$ and $p_{\mathrm{adj}} < 0.01$.

A

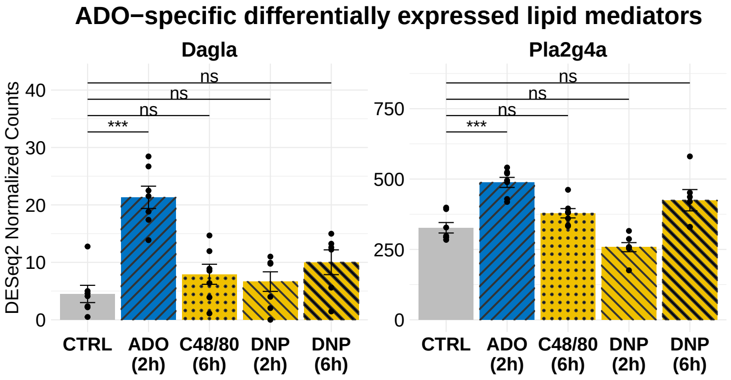
ADO−specific differentially expressed lipid mediators
Dagla
Pla2g4a
DESeq2 Normalized Counts
0
10
20
30
40
0
250
500
750
ns
ns
ns
***
CTRL
ADO (2h)
C48/80 (6h)
DNP (2h)
DNP (6h)

B

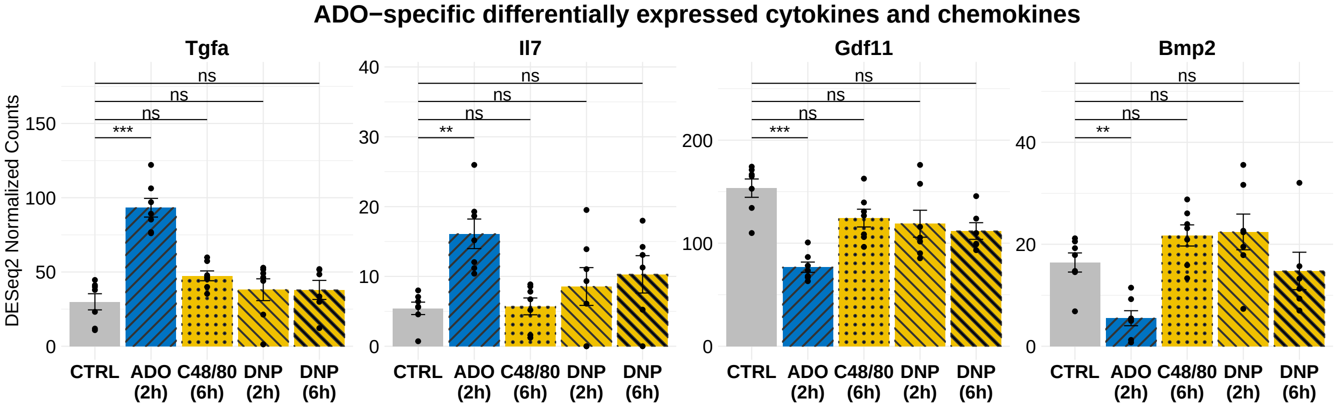
ADO−specific differentially expressed cytokines and chemokines
Tgfa
Il7
Gdf11
Bmp2
DESeq2 Normalized Counts
0
50
100
150
0
10
20
30
40
0
100
200
0
20
40
ns
ns
ns
***
**
CTRL
ADO (2h)
C48/80 (6h)
DNP (2h)
DNP (6h)

C

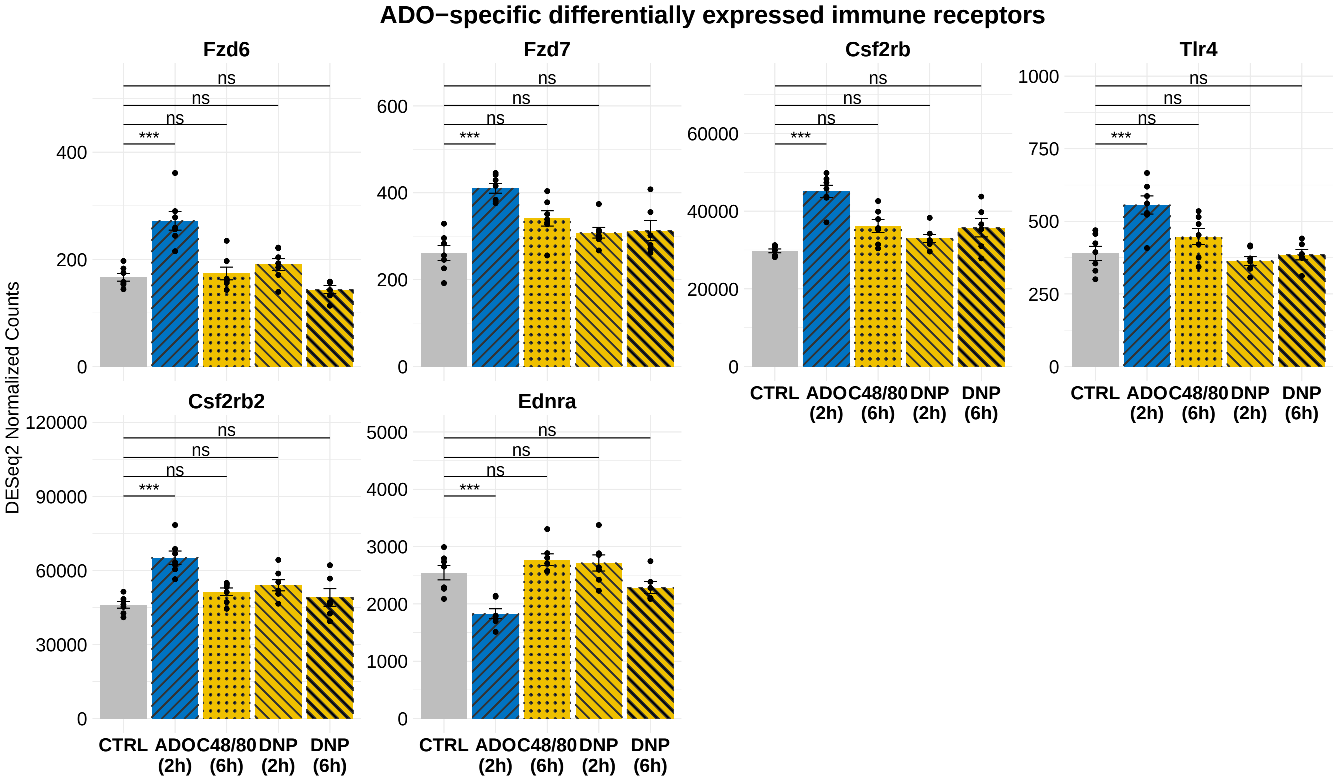
ADO−specific differentially expressed immune receptors
Fzd6
Fzd7
Csf2rb
Tlr4
Csf2rb2
Ednra
DESeq2 Normalized Counts
0
200
400
0
200
400
600
0
20000
40000
60000
0
250
500
750
1000
0
30000
60000
90000
120000
0
1000
2000
3000
4000
5000
ns
ns
ns
***
CTRL
ADO (2h)
C48/80 (6h)
DNP (2h)
DNP (6h)

D

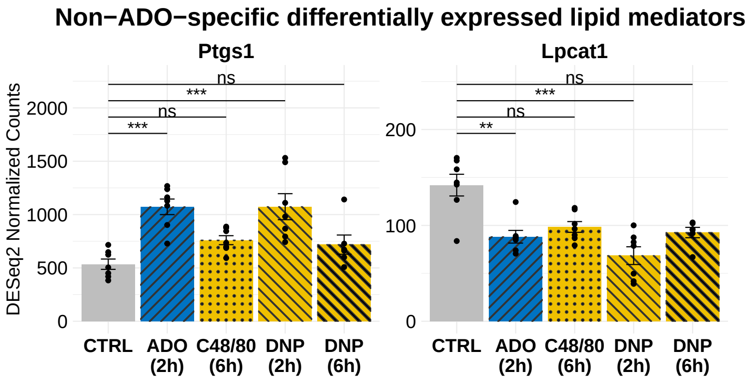
Non−ADO−specific differentially expressed lipid mediators
Ptgs1
Lpcat1
DESeq2 Normalized Counts
CTRL
ADO (2h)
C48/80 (6h)
DNP (2h)
DNP (6h)
ns
***
**

E

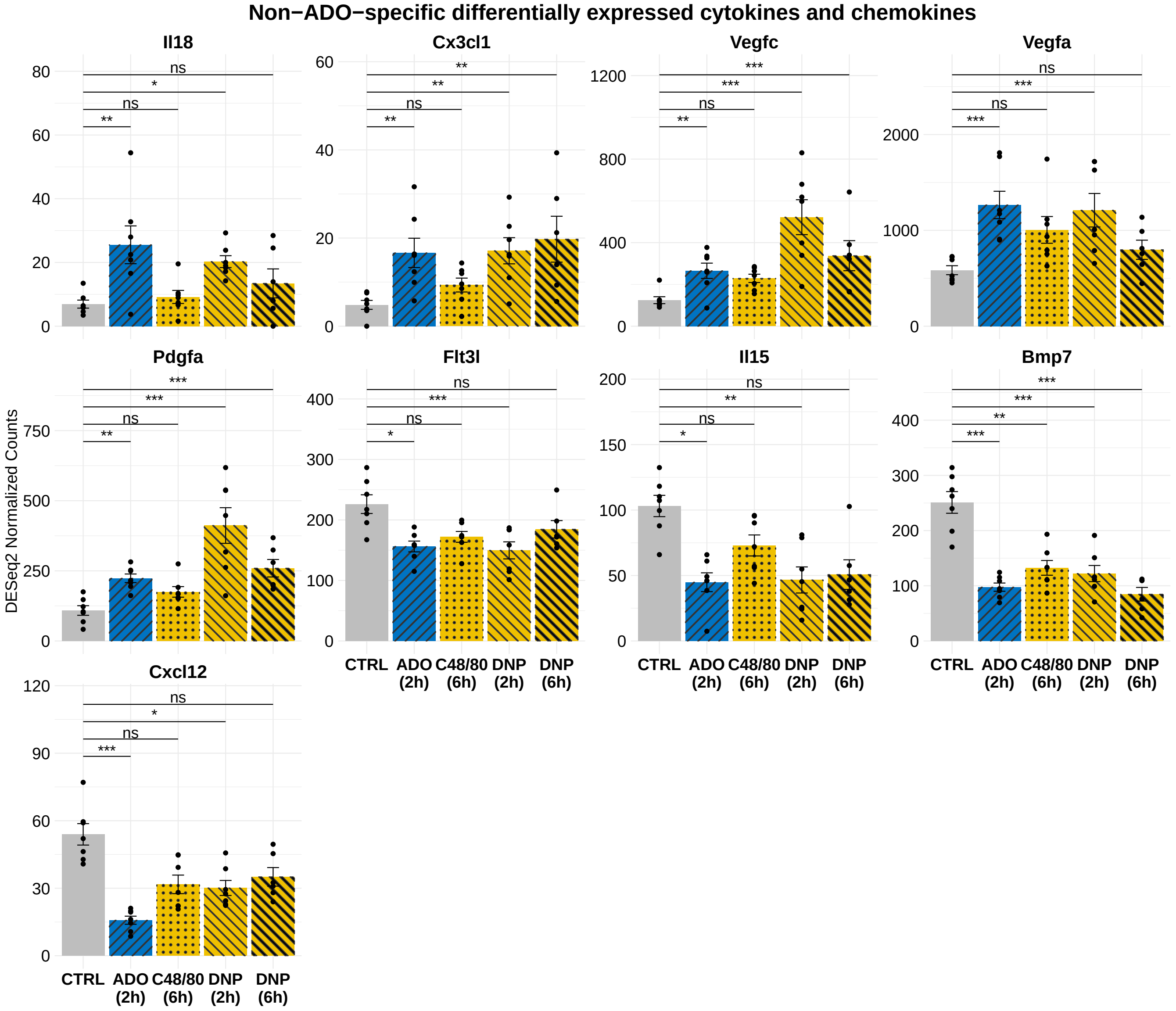
Non−ADO−specific differentially expressed cytokines and chemokines
Il18
Cx3cl1
Vegfc
Vegfa
Pdgfa
Flt3l
Il15
Bmp7
Cxcl12
DESeq2 Normalized Counts
CTRL
ADO (2h)
C48/80 (6h)
DNP (2h)
DNP (6h)
ns
*
**
***

F

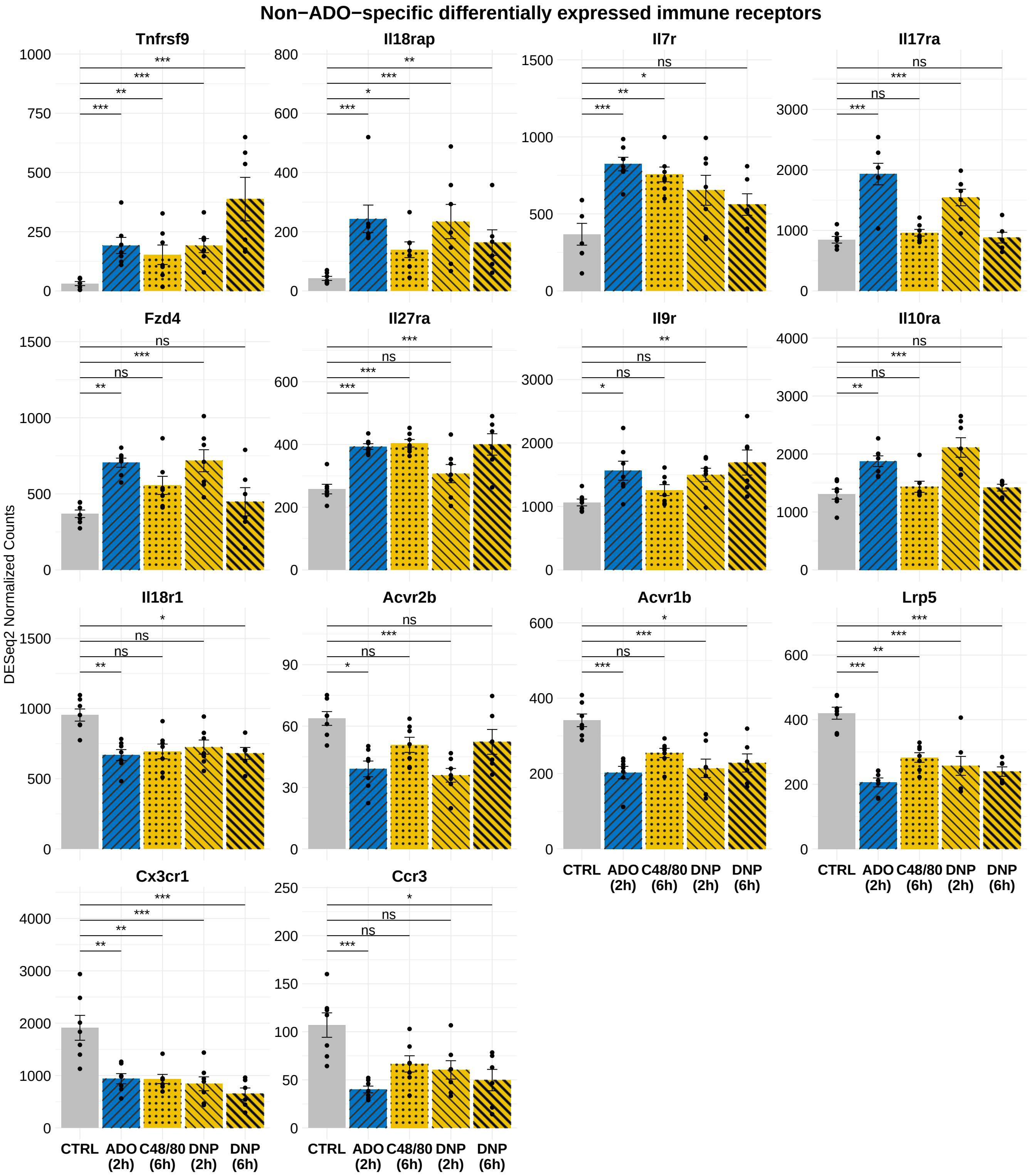

Figure S4: Expression profiling of inflammatory mediators regulated by ADO in PMCs.
(A) Lipid mediators, **(B)** cytokines, chemokines, or growth factors, and **(C)** cytokine or chemokine receptors that were significantly and exclusively regulated by ADO. **(D)** Lipid mediators, **(E)** cytokines, chemokines, or growth factors, and **(F)** cytokine or chemokine receptors that were significantly, but not exclusively, regulated by ADO. Genes were sorted in descending order according to $\log_2$ FC. Statistical significance was assessed using DESeq2, with significance levels defined as follows: ns, not significant; $*p_{\text{adj}} < 0.01$; $**p_{\text{adj}} < 0.005$; $***p_{\text{adj}} < 0.001$.

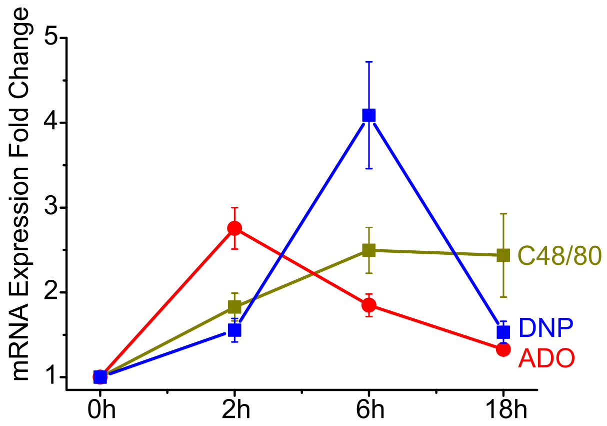

Figure S5: Time course of the mean fold-change (normalised to untreated group (0h)) of mRNA expression level of 36 genes of interest*. Conditions include untreated (0h), 2 hours of treatment (2h), 6 hours of treatment (6h), 18 hours of treatment (18h) with 10 µM of adenosine (red line & points), with 50 µg/mL of C48/80 (dark-yellow line & points) and with 100 ng/mL of antigen (blue line & points). N=1 (biological replicates); n=1 (technical replicates); the error bars represent SEM; *(Il18rap, Igsf3, Nrip3, Scarb1, Lonp1, Retreg1, Cdkn1a, Peak1, Ttc21b, Mpp6, Lmna, Gsdme, Psat1, Runx2, Ptpn3, Prrg4, Fam110c, Nab2, Myc, Serpinb1a, Cd44, Dapp1, Vegfc, Trank1, Mboat2, Gpr141b, Atp13a3, Bmp7, Ikzf4, Sdr39u1, Cdk14, Mrgprx1, Gpx3, Steap3, Klra2, Cgnl1).

Table S1: DESeq2 normalized expression of calcium ion channels in CONTROL, ADO (2h), C48/80 (6h), DNP (2h), and DNP (6h)-treated samples. Genes that are differentially expressed compared to the CONTROL are marked with asterisks: * for $p_{\text{adj}} < 0.05$, ** for $p_{\text{adj}} < 0.01$, and *** for $p_{\text{adj}} < 0.001$.

Gene Name	CONTROL	ADO (2h)	C48/80 (6h)	DNP (2h)	DNP (6h)
**Voltage-gated calcium channel $\alpha 1$ subunits (CACNA1 family)**					
Cacna1s	1.0	1.5	1.9	1.3	1.8
Cacna1c	7.0	7.2	6.9	6.5	7.8
Cacna1d	41.9	54.2	43.2	39.1	36.1
Cacna1f	1.5	0.3	1.9	0.9	0.4
Cacna1a	16.7	13.8	14.8	11.4	13
Cacna1b	3.2	2.6	2.3	1.6	2.1
Cacna1e	54.1	54.6	52.4	33.6	33
Cacna1g	0.5	0.9	0.4	0	0
Cacna1h	54.5	72.9	82.4	108.3	103.7
Cacna1i	25.4	18.5	22.1	18.4	15.2
**Voltage-gated calcium channel auxiliary β subunits (CACNB family)**					
Cacnb1	10.9	7.1	7.5	12	8.9
Cacnb2	10.4	9.1	8.2	10.2	12.2
Cacnb3	7.7	7.5	8.4	8.8	9.8
Cacnb4	261.7	263.4	188.2 *	248	135.2 **
**Voltage-gated calcium channel auxiliary $\alpha 2\delta$ subunits (CACNA2D family)**					
Cacna2d1	4.1	5.6	10.8	2.5	4.9
Cacna2d2	0.6	0.7	0.7	2.7	0.9
Cacna2d3	0.0	0	0	1	0.1
Cacna2d4	16.8	10.9	13.5	16.3	10.1
**Voltage-gated calcium channel auxiliary γ subunits (CACNG family)**					
Cacng1	0.1	0.1	0	0	0.1
Cacng2	0.0	0	0.3	0	0
Cacng3	2.8	1.1	1.3	0.3	3.2
Cacng4	0.1	0	0.2	0	0
Cacng5	0.3	0	0.2	0	0.1
Cacng6	0.6	0.1	0.5	0	0.3
Cacng7	241.2	181.7	173.4 *	200.8	137.2 ***
Cacng8	44.3	24.4 *	25.8	26.5 *	24.7 *
**Ryanodine receptors (RYR family)**					
Ryr1	2.0	1.5	3.1	1.2	0.9
Ryr2	0.7	0.8	1.4	0.3	0.4
Ryr3	6112.1	5642.1	9030.5 ***	6649.1	9497.6 ***
**Inositol 1,4,5-trisphosphate receptors (ITPR family)**					
Itpr1	1683.7	1386.7 *	1461.1	1608	1408.2 **
Itpr2	4395.9	4008.7	4076.6	3894.7	3682.3 **
Itpr3	547.7	551.3	652.4	540.6	791.7 **

Continued on next page

Table S1 – continued from previous page

Gene Name	CONTROL	ADO (2h)	C48/80 (6h)	DNP (2h)	DNP (6h)
**Lysosomal calcium channels**					
Tpcn1	247.9	226.3	187.4 *	204.6	206.9
Tpcn2	122.8	114.9	120.8	114.6	112.9
Tmem63a	325.5	279.7	258.6 *	269.4	257.6 *
Tmem63b	141.5	199 *	230.8 **	224.1 **	279.4 ***
**Transient receptor potential channels**					
Trpc1	0.8	2.2	1.3	2.2	1.1
Trpc2	6.4	4.1	3.4	1.6	7.7
Trpc3	0.3	0.1	0.2	0.4	0
Trpc4	0.9	0.8	0.2	0.8	0.1
Trpc5	4.9	2.4	4.5	2.5	2.8
Trpc6	12.4	6.5	7.8	3.6 **	3.9 **
Trpc7	45.2	30.9	23.7	31.6	15.7 **
Trpv1	1.7	0.4	0.2	0.2	0.4
Trpv2	212.3	245.4	260.1	265.9	166.1
Trpv3	0.2	0	0	0	0.2
Trpv4	2.4	3.8	3.7	1.1	1.2
Trpv5	0.0	0	0	0	0
Trpv6	0.4	0.4	0.1	0	0
Trpm1	0.1	0.1	0.1	0	0
Trpm2	113.4	83.7	65.6 *	94.7	61.4 *
Trpm3	2.4	2.5	2.7	1.8	0.7
Trpm4	267.6	459.7 **	252.1	498.9 ***	375.3
Trpm5	2.1	1.6	2.7	3.1	1.4
Trpm6	0.0	0.2	0.2	0	0.1
Trpm7	1721.6	1682.6	1682.3	1759.4	1815.9
Trpm8	0.0	0	0.1	0	0
Trpa1	0.0	0	0.2	0	0
Mcoln1	377.0	303.5 *	335	322.7	389.4
Mcoln2	33.2	30.5	35.3	25.9	35.2
Mcoln3	1.3	0.5	0.8	1.5	2.5
Pkd2	412.1	376.4	364	378.5	368.8
Pkd2l1	0.0	0	0	0	0
Pkd2l2	65.7	58.2	42.2	62.6	49.9
**Store-Operated Calcium Entry (SOCE)**					
Orai1	114.3	161.4	142.2	223.7 ***	125.8
Orai2	576.5	494.8	552	519.2	503.1
Orai3	247.5	227.9	242.3	194.4 **	209.5
Stim1	1526.0	1048.1 ***	1295	1180.8 **	1101.2 ***
Stim2	491.7	502.9	487	703.9 ***	522.9

Continued on next page

Table S1 – continued from previous page

Gene Name	CONTROL	ADO (2h)	C48/80 (6h)	DNP (2h)	DNP (6h)
**P2X receptors (ATP-gated)**					
P2rx1	589.1	569.8	576.5	752.8 ***	534.9
P2rx2	0.3	0.2	0.3	0.2	0.3
P2rx3	0.8	1.9	1.6	1.3	1.4
P2rx4	2611.5	2091.5 *	1879.2 **	1959.5 **	1994 *
P2rx5	7.8	10.5	9.2	13.4	8.5
P2rx6	4.0	4.6	3.6	1.5	2.1
P2rx7	2596.6	1937.1 *	1966.2 *	2117.6 *	1974.7 *
**Piezo family**					
Piezo1	230.4	371.9	346.6	1646.3 ***	506.7 *
Piezo2	6.1	6.4	29.6	9.6	6.7
**NMDA receptors**					
Grin1	0.0	0.5	0	0	0
Grin2a	0.0	0.4	0	0	0
Grin2b	0.0	0	0	0	0.2
Grin2c	307.0	200.6 ***	164.4 ***	277.5	191.2 **
Grin2d	539.3	274.6 ***	383.2	469.3	420.2 *
Grin3a	26.0	23.7	22.5	24.8	22.7
Grin3b	3.7	3.4	3.5	4.8	2.9
**AMPA receptor channel subunits**					
Gria1	1.5	1	1.3	1.1	3
Gria2	0.3	0	0	0.2	0
Gria3	19.8	20.4	33.2	23.4	22
Gria4	0.3	0.1	0.3	0	0.5
**Calcium homeostasis modulator**					
Calhm1	0.1	0.1	0	0.3	0
Calhm2	526.7	332.9 **	381.9	361.5 **	393.9
Calhm3	0.0	0	0	0	0
Calhm4	0.0	0	0	0	0.1
Calhm5	0.1	0.5	0.1	0	0
Calhm6	0.4	0.9	0.5	0.4	0.7

Table S2: Differential expression statistics of all ADO-specific protein coding genes. Genes were ordered in terms of their normalized expression value in either CONTROL or ADO 2 hour-treated condition.

Gene Name	CONTROL	ADO (2h)	log2FC	padj
Csf2rb2	46038.7	65174.7	0.5	2.0e-05
Csf2rb	29766.2	45078.7	0.6	3.5e-08
Gata2	14346.1	10470.5	-0.5	1.4e-05
Bod1l	5279.0	9133.9	0.8	7.1e-05
Ywhaz	6013.5	8442.7	0.5	3.2e-05
Mtss1	7503.2	5092.1	-0.6	5.5e-10
Aldoa	5709.2	7296.7	0.4	4.1e-03
Bltp1	4509.3	7196.8	0.7	3.7e-06
Notch2	5695.8	7134.9	0.3	7.8e-03
Rab27b	4326.2	6951.7	0.7	7.6e-06
Niban1	2912.3	6381.1	1.1	2.3e-15
Zbtb38	3234.7	5941.0	0.9	1.9e-16
Prkar1a	3784.5	4831.1	0.3	3.2e-03
Ikzf2	4658.4	3452.4	-0.4	7.4e-03
Ldha	2525.0	4618.7	0.8	9.8e-11
Pfkp	2521.4	4204.5	0.7	8.1e-08
Ubash3b	4110.8	2851.0	-0.5	3.5e-08
Paxbp1	2472.8	4110.2	0.7	9.7e-05
Sdcbp	2862.8	3910.0	0.5	3.8e-04
Trio	2338.3	3815.6	0.7	5.4e-06
Rfx7	3637.1	2698.2	-0.4	2.7e-06
Itch	3491.3	2557.6	-0.4	3.1e-07
Fryl	3477.7	2860.3	-0.3	2.3e-04
Arrb1	2578.0	3396.6	0.4	5.9e-06
Otulinl	2299.3	3383.4	0.5	3.7e-04
Ston2	1846.1	3371.9	0.8	3.7e-04
Gvin1	1830.2	3052.5	0.7	5.3e-03
Tnpo1	2405.6	2823.8	0.2	8.7e-03
Prkcd	2804.9	2089.5	-0.4	2.8e-04
Vat1	2096.1	2785.4	0.4	3.2e-03
Taok3	2759.7	2317.6	-0.3	6.6e-04
Gvin2	1314.7	2701.0	1.0	2.8e-03
Myo5a	2190.1	2615.1	0.3	8.0e-03
Ptpn13	1260.8	2589.0	1.0	4.7e-09
Ednra	2544.4	1827.7	-0.5	9.1e-05
Mrgprx2	1619.1	2460.4	0.6	3.7e-03
Sema4d	2452.8	1673.0	-0.5	3.3e-04
Arid4b	2354.4	1794.7	-0.4	2.0e-04
Plcg1	2337.5	1644.4	-0.5	1.0e-06

Continued on next page

Table S2 – continued from previous page

Gene Name	CONTROL	ADO (2h)	log2FC	padj
Mat2a	2322.7	1746.7	-0.4	8.9e-03
Gab2	1651.4	2322.5	0.5	4.5e-03
Top1	2239.5	1618.0	-0.5	2.1e-03
Tns3	2140.6	1512.9	-0.5	3.2e-05
Arap1	2009.3	1420.0	-0.5	8.1e-07
Sipa1l1	1113.4	1936.3	0.8	6.1e-05
Gmfb	1603.4	1918.7	0.3	5.6e-03
Nckap1	1862.9	1456.6	-0.4	5.2e-13
Cic	1819.1	1458.2	-0.3	5.8e-04
Hs6st1	1055.1	1783.7	0.7	3.3e-04
Ap1g1	1380.7	1682.1	0.3	9.0e-04
Nup153	1678.0	1368.7	-0.3	2.8e-03
Mau2	1352.9	1669.4	0.3	4.9e-04
Fermt3	1631.7	1322.0	-0.3	3.5e-06
Tiparp	1592.6	978.6	-0.7	4.6e-04
Serpinb6a	1126.3	1573.2	0.5	2.3e-04
Nrdc	1378.3	1571.9	0.2	8.1e-03
Acap2	1460.6	1203.2	-0.3	1.9e-03
Ccser2	1154.4	1449.5	0.3	8.3e-04
Coro1c	1125.9	1443.3	0.4	2.3e-03
P4ha1	1412.2	1142.9	-0.3	1.3e-05
Arpc3	905.2	1403.6	0.6	1.8e-06
Isy1	284.8	1371.3	2.2	1.3e-14
Arhgap25	1052.2	1355.5	0.4	1.5e-03
Srpk1	1349.8	1151.0	-0.2	3.1e-03
Phldb3	891.9	1338.7	0.6	2.5e-05
Nceh1	946.7	1303.3	0.5	3.6e-03
Kif13b	1291.2	965.7	-0.4	2.8e-03
Gga2	695.6	1272.8	0.9	9.2e-08
Crem	142.8	1265.5	3.1	2.3e-15
Tmed5	869.9	1248.7	0.5	6.2e-03
Lxn	767.1	1244.4	0.7	2.8e-07
Dstyk	1033.3	1232.7	0.3	5.7e-03
Coro2a	1216.7	702.6	-0.7	6.1e-03
Itk	495.5	1198.1	1.2	6.3e-04
Gak	979.7	1191.1	0.3	4.0e-03
Pou2f1	896.0	1187.8	0.4	4.1e-03
Plekha2	569.0	1180.3	1.1	2.0e-09
Slc2a1	1162.1	808.9	-0.5	2.3e-03
Dctn4	881.9	1145.2	0.4	2.0e-04
Mbtps1	1131.0	980.4	-0.2	9.1e-03

Continued on next page

Table S2 – continued from previous page

Gene Name	CONTROL	ADO (2h)	log2FC	padj
Map2k4	937.6	1122.9	0.3	9.5e-03
Gpbp1l1	1096.3	938.6	-0.2	8.9e-03
Nedd4l	704.3	1014.6	0.5	4.1e-03
Sbno2	748.1	1012.3	0.5	1.1e-05
Slc25a5	644.7	1004.5	0.6	2.7e-04
Stat4	656.7	1001.8	0.6	2.0e-03
St3gal2	989.3	667.0	-0.6	3.0e-03
Rap2b	466.8	972.7	1.1	2.0e-16
Cd300lb	456.7	946.3	1.1	1.0e-07
Srek1	943.5	758.7	-0.3	7.8e-03
Lrrc1	619.9	943.0	0.6	1.3e-05
Ttpal	671.6	936.7	0.5	2.6e-03
Vamp4	632.0	930.4	0.6	1.5e-10
Flcn	922.0	768.0	-0.3	7.9e-03
Pmp22	485.8	918.2	0.9	2.8e-07
Zfp398	764.8	906.9	0.3	3.8e-03
Kpna1	618.3	880.4	0.5	5.8e-04
Osbpl9	580.5	873.2	0.6	1.3e-05
Jade1	643.2	872.9	0.4	2.6e-04
Wdr81	872.5	672.1	-0.4	9.6e-04
Elovl5	662.2	865.2	0.4	5.2e-03
Ntng2	396.7	851.1	1.1	2.8e-04
Wbp2	846.4	636.9	-0.4	1.7e-03
Klf10	478.7	841.4	0.8	2.2e-06
Med14	605.5	830.1	0.4	2.9e-03
Kdm4b	827.9	637.1	-0.4	8.5e-03
Nap1l5	219.3	821.2	1.9	1.5e-09
Tpm4	335.6	811.8	1.2	1.0e-05
Sntb2	578.4	802.5	0.5	1.1e-04
Amph	463.5	791.6	0.8	4.3e-03
Mpp7	77.1	781.3	3.4	3.1e-38
Cpt1a	600.0	780.1	0.4	9.0e-04
Slc36a4	661.5	777.4	0.2	5.6e-03
Scin	476.3	763.2	0.7	5.2e-03
Dhrs3	760.0	445.4	-0.8	6.9e-03
Card11	747.7	389.0	-0.9	2.3e-08
Pitpnc1	565.8	747.6	0.4	7.5e-03
Galc	415.1	727.3	0.8	2.9e-06
Plin2	415.9	722.9	0.8	8.9e-05
Rrp1	568.6	719.7	0.3	4.7e-03
Pik3cb	524.8	713.9	0.4	9.8e-04

Continued on next page

Table S2 – continued from previous page

Gene Name	CONTROL	ADO (2h)	log2FC	padj
Ric1	709.8	548.8	-0.4	5.4e-04
Gusb	474.4	701.6	0.5	2.2e-03
Aida	537.0	691.3	0.4	6.3e-04
Sap130	690.1	560.5	-0.3	3.5e-03
Limd2	682.8	484.8	-0.5	3.1e-05
Qser1	543.4	680.0	0.3	9.8e-03
Calcoco1	678.3	487.9	-0.5	1.2e-03
Rfk	482.0	671.2	0.5	6.9e-04
Ulk1	662.6	485.7	-0.4	8.2e-03
Rfx5	648.4	511.4	-0.3	2.7e-04
Pi4kb	470.6	641.8	0.4	1.1e-03
Mink1	635.9	430.1	-0.6	3.5e-05
Ctla2b	354.1	622.4	0.8	1.3e-04
Lrch4	612.0	375.2	-0.7	2.7e-03
Stx7	517.2	598.3	0.2	6.4e-03
Vdac3	493.7	597.8	0.3	3.5e-03
Pts	465.8	593.5	0.4	3.2e-03
Man1b1	443.9	588.7	0.4	1.3e-03
Tbcel	585.4	488.3	-0.3	7.7e-03
Disp1	361.8	574.2	0.7	2.4e-10
Entpd7	355.6	559.4	0.6	3.8e-07
Tlr4	389.8	556.2	0.5	5.2e-04
Rnf217	355.8	535.5	0.6	1.4e-06
Cemip2	520.8	321.7	-0.7	5.9e-04
Golt1b	415.9	519.8	0.3	5.1e-03
Antxr2	329.3	516.2	0.7	9.8e-03
Strap	374.6	512.3	0.5	2.9e-03
Mcur1	501.2	411.4	-0.3	3.5e-03
Rassf3	345.8	496.9	0.5	4.7e-03
Rhoq	333.5	496.0	0.6	7.2e-03
Grin2d	495.1	244.8	-1.0	9.8e-06
Ninj1	261.5	493.8	0.9	1.6e-07
Asb1	489.0	353.0	-0.5	7.8e-04
Unc45a	378.0	488.8	0.4	1.5e-03
Pla2g4a	326.9	488.1	0.6	3.6e-04
Emd	245.6	487.5	1.0	5.5e-14
Plekha5	297.3	481.2	0.7	2.5e-04
Gucd1	365.5	479.1	0.4	5.2e-03
Ksr1	474.0	291.8	-0.7	1.8e-04
Adam9	353.0	472.6	0.4	6.2e-03
Hpcal1	142.8	470.0	1.8	7.6e-12

Continued on next page

Table S2 – continued from previous page

Gene Name	CONTROL	ADO (2h)	log2FC	padj
Slc2a6	467.8	294.3	-0.6	3.1e-06
Tmem184c	466.9	358.0	-0.4	8.0e-03
Pias2	466.0	398.2	-0.2	7.8e-03
Prune1	360.3	465.7	0.4	5.6e-03
Atp5f1d	327.7	456.1	0.5	2.1e-03
Inip	316.3	451.5	0.5	7.2e-04
Wdr44	362.1	450.6	0.3	4.2e-03
Smim30	358.0	450.0	0.3	2.0e-03
Map4k2	449.5	327.9	-0.5	7.1e-03
Dgka	449.3	330.1	-0.5	4.0e-03
Ptch1	435.2	308.0	-0.5	1.0e-04
Fbxo9	250.1	432.6	0.8	8.9e-17
Gata1	431.4	247.4	-0.8	9.9e-04
Eif1ax	326.7	425.5	0.4	1.8e-03
Zeb1	423.7	321.5	-0.4	2.2e-03
Ncoa7	416.0	260.2	-0.7	2.3e-09
Nol4l	415.2	257.9	-0.7	1.1e-04
Tbc1d31	287.9	414.5	0.5	4.7e-03
Fzd7	261.0	410.4	0.6	6.7e-06
Bcl9	405.9	250.0	-0.7	7.2e-09
Prep	254.1	396.4	0.6	5.4e-04
Txndc5	298.9	389.5	0.4	6.6e-03
Lztfl1	311.0	388.0	0.3	7.6e-04
Plcb3	387.2	254.6	-0.6	1.7e-06
Zcchc10	383.8	213.1	-0.8	4.8e-05
Nfix	383.3	289.8	-0.4	3.6e-03
Pcgf5	249.5	370.4	0.6	4.6e-04
Slc6a12	364.4	231.7	-0.7	2.3e-03
Tesk2	363.2	246.0	-0.6	6.5e-04
Ccnc	275.9	359.6	0.4	2.8e-03
Rbm45	212.1	350.8	0.7	1.6e-05
Eeig1	350.2	212.4	-0.7	6.3e-04
Osbpl6	28.2	346.2	3.7	1.6e-09
Apex2	340.9	244.1	-0.5	5.5e-03
Fcho1	336.7	275.2	-0.3	8.0e-03
Ptov1	335.0	257.4	-0.4	6.7e-03
Slc25a37	233.8	329.7	0.5	4.8e-04
Tshz3	328.8	214.2	-0.6	6.2e-06
Impdh1	216.0	318.0	0.5	1.8e-04
Bcorl1	315.8	206.4	-0.6	7.0e-05
Pgpep1	312.7	201.6	-0.6	9.4e-05

Continued on next page

Table S2 – continued from previous page

Gene Name	CONTROL	ADO (2h)	log2FC	padj
Mfhas1	208.8	312.7	0.6	2.7e-04
Slc20a2	224.7	311.3	0.5	9.7e-03
Osgin1	305.1	167.6	-0.8	3.7e-03
Sdhb	226.2	303.5	0.4	2.0e-04
Tinf2	206.5	290.2	0.5	3.4e-04
Ccdc167	288.4	221.0	-0.4	6.5e-03
Rnf157	288.2	206.4	-0.5	1.1e-03
Bbs12	199.9	284.0	0.5	2.7e-03
Cyth1	196.1	275.9	0.5	3.2e-03
Ap5z1	273.7	206.7	-0.4	9.0e-03
Smg9	215.5	272.3	0.3	6.1e-03
Fzd6	166.5	272.0	0.7	1.5e-06
Nfkbie	269.6	187.1	-0.5	4.0e-03
Aph1c	158.9	268.2	0.8	2.4e-06
Phlpp2	266.0	191.9	-0.5	2.8e-03
Tirap	150.4	263.8	0.8	7.1e-09
Hdac7	263.8	183.7	-0.5	9.5e-04
Zfp956	145.6	261.7	0.9	8.7e-12
Nsun4	160.0	254.5	0.7	4.1e-07
Rabep2	251.1	181.7	-0.5	1.4e-03
Unc119b	198.2	249.3	0.3	1.2e-03
Grm5	106.9	244.7	1.2	3.9e-03
Tmc4	146.0	243.2	0.7	7.0e-03
Crtc1	242.7	183.1	-0.4	8.2e-03
Lactb2	180.8	241.1	0.4	1.1e-03
Plxnb3	239.6	125.0	-1.0	3.0e-03
Zfhx2	238.7	165.3	-0.6	4.6e-03
Lrrc57	238.4	160.3	-0.6	1.7e-06
Card19	158.8	238.3	0.6	6.6e-03
Ahdc1	236.0	141.7	-0.7	5.5e-03
Mfsd12	119.8	232.3	1.0	7.6e-08
Tpbgl	106.5	231.5	1.1	7.0e-04
Dlg4	231.3	159.1	-0.5	7.8e-05
Arf2	143.4	230.8	0.7	7.3e-04
Lmnb1	82.9	216.8	1.3	3.0e-05
Kcnn4	135.2	216.7	0.7	1.1e-03
Gcsh	74.3	208.7	1.5	3.9e-12
Stk39	208.3	151.2	-0.4	2.8e-03
Slc16a3	143.6	207.8	0.5	1.4e-03
Mpped2	204.0	150.2	-0.4	5.3e-03
Wdr90	99.2	201.2	1.0	1.9e-05

Continued on next page

Table S2 – continued from previous page

Gene Name	CONTROL	ADO (2h)	log2FC	padj
Zcchc14	154.8	200.8	0.4	2.4e-03
Dgke	147.8	197.3	0.4	8.1e-03
Rab4a	56.3	194.3	1.8	1.9e-16
Usp20	191.9	134.7	-0.5	2.3e-03
Zfp619	191.2	139.0	-0.5	7.7e-03
Klhl23	132.0	189.6	0.5	2.3e-03
Aatk	187.5	69.4	-1.4	2.7e-05
Axin2	187.4	130.6	-0.5	6.7e-03
Pnkd	180.3	107.5	-0.7	1.7e-03
Insig1	178.8	103.3	-0.8	1.3e-03
Ifih1	126.0	178.5	0.5	1.7e-03
Uchl3	175.3	93.8	-0.9	1.2e-05
Cnnm2	35.8	174.9	2.3	8.6e-08
Cxxc5	174.3	111.0	-0.6	6.5e-03
Gpr63	112.5	169.5	0.6	8.5e-04
Llgl1	167.6	116.8	-0.5	2.2e-03
Cnrip1	165.6	117.7	-0.5	9.5e-03
Thumpd2	165.3	97.2	-0.8	6.5e-03
Slco3a1	22.6	161.2	2.9	7.5e-14
Ggcx	158.3	119.9	-0.4	5.9e-03
Chaf1b	156.6	102.7	-0.6	4.6e-03
Ubtd1	106.7	156.4	0.6	3.2e-04
Gdf11	153.5	76.7	-1.0	8.1e-08
Arrdc4	72.4	151.4	1.1	1.5e-07
Hdac6	151.1	95.1	-0.7	7.0e-04
Gm21190	149.5	69.4	-1.1	3.4e-04
Glis2	147.9	77.5	-0.9	3.1e-03
Cbarp	63.3	145.2	1.2	6.1e-07
Pfkfb4	141.3	94.8	-0.6	3.7e-03
Mrm1	101.1	139.0	0.5	3.4e-03
Rarb	55.0	138.5	1.4	3.9e-07
Pold1	138.0	89.2	-0.6	4.8e-03
Ears2	52.0	137.3	1.4	1.3e-05
Atp5mk	89.4	135.5	0.6	5.8e-03
Klhl25	95.5	134.7	0.5	1.6e-03
Abhd11	130.9	92.0	-0.5	8.8e-03
Rab11fip5	77.5	125.4	0.7	2.1e-03
Sestd1	87.4	124.6	0.5	3.2e-03
Gpr85	80.9	120.8	0.6	4.0e-03
Bcar3	116.3	52.9	-1.1	4.6e-03
Osbpl1a	113.6	80.7	-0.5	7.1e-03

Continued on next page

Table S2 – continued from previous page

Gene Name	CONTROL	ADO (2h)	log2FC	padj
Nckap5l	111.4	73.9	-0.6	6.2e-03
Rgl3	107.8	70.9	-0.6	5.9e-03
Sh2b2	39.5	104.0	1.4	2.8e-05
Hes1	98.8	36.3	-1.5	2.3e-03
Qsox2	62.1	98.1	0.6	4.1e-03
Pcgf2	98.0	53.8	-0.9	9.4e-05
Ano8	96.4	67.5	-0.5	6.4e-03
Igsf5	16.5	95.0	2.5	3.4e-12
Focad	93.7	62.2	-0.6	3.9e-03
Tgfa	29.9	93.3	1.7	1.4e-05
Pde10a	23.2	86.7	1.9	2.2e-04
Pdlim1	32.4	83.7	1.4	8.5e-04
Snn	82.3	51.8	-0.7	8.6e-03
Kcnd1	80.5	47.6	-0.8	8.9e-03
Klra2	36.5	76.8	1.1	1.2e-04
Polg2	75.5	50.4	-0.6	9.7e-03
Slc35f3	74.8	44.0	-0.7	7.1e-03
Ccsap	54.2	73.1	0.5	5.3e-03
P2ry10	29.0	71.6	1.3	2.6e-03
Mcm10	70.9	43.1	-0.7	2.3e-03
Zfp248	70.1	40.0	-0.8	4.2e-03
Snx7	66.8	47.2	-0.5	2.4e-03
Slc46a1	23.5	64.9	1.5	7.3e-05
C1qtnf12	25.6	61.6	1.2	3.7e-04
Gm20696	56.2	24.6	-1.2	1.6e-04
Msi1	54.5	29.1	-0.9	4.8e-03
Ncaph	53.9	30.0	-0.8	5.5e-03
Lrrc36	53.7	18.4	-1.6	3.8e-03
Hoxb4	51.9	32.9	-0.6	8.0e-03
Sdhaf4	49.9	29.1	-0.8	9.1e-03
Atp8b1	48.8	20.8	-1.2	3.3e-04
Draxin	20.8	46.6	1.2	1.1e-05
Fancd2	45.9	27.8	-0.7	6.4e-03
Twist2	45.3	15.8	-1.6	1.5e-03
Dennd5b	43.5	19.1	-1.2	7.3e-03
Rnf227	42.9	24.0	-0.9	1.7e-04
Lpar2	26.4	42.6	0.7	6.1e-03
Hid1	13.3	42.3	1.7	9.6e-04
Whrn	13.0	41.9	1.7	7.4e-04
Frmd5	16.4	41.0	1.3	2.7e-03
Sap30	18.9	36.9	0.9	4.3e-03

Continued on next page

Table S2 – continued from previous page

Gene Name	CONTROL	ADO (2h)	log2FC	padj
Hfe	14.7	36.6	1.3	1.0e-03
Micall2	12.4	35.0	1.5	1.0e-03
Mettl15	34.5	20.8	-0.7	8.8e-03
Nphp4	9.0	34.4	2.0	6.5e-05
Grhpr	32.6	15.4	-1.1	5.8e-03
Slc25a35	29.1	12.1	-1.2	5.5e-03
Tifa	27.7	13.3	-1.2	5.2e-03
Lrrc2	11.1	26.9	1.3	7.3e-03
Samd11	7.7	26.1	1.7	3.0e-03
Rwdd2a	25.8	8.3	-1.7	1.4e-04
Or2y1f	22.5	4.2	-2.4	1.8e-04
Dagla	4.5	21.3	2.3	1.5e-04
Gng4	6.1	20.2	1.8	3.1e-03
Rln3	1.3	17.3	4.0	1.4e-06
Fndc9	16.8	6.0	-1.2	3.6e-03
Bmp2	16.4	5.5	-1.5	2.6e-03
Il7	5.4	16.1	1.4	3.0e-03
Apol9b	16.1	4.7	-1.9	6.7e-03
Itgb6	15.8	5.6	-1.7	4.5e-03
Me3	4.1	15.4	1.8	1.6e-03
Tmem202	5.6	14.1	1.4	5.4e-04
Trim58	12.1	2.4	-2.3	1.6e-05
Kcns3	10.7	2.3	-2.2	1.6e-04

Table S3: Summary of top six most abundant protein classes among upregulated ADO-specific genes.

Protein Classes	Associated Genes
**metabolite interconversion enzyme (PC00262)**	
acyltransferase(PC00042)	Elovl5 Cpt1a
aldolase(PC00044)	Aldoa
carbohydrate kinase(PC00065)	Pfkp
deacetylase(PC00087)	Nceh1
dehydrogenase(PC00092)	Impdh1 Sdhb Ldha
galactosidase(PC00104)	Galc
guanylate cyclase(PC00114)	Gucd1
kinase(PC00137)	Pik3cb Dgke Pi4kb
lipase(PC00143)	Dagla Golt1b
nucleotide phosphatase(PC00173)	Entpd7
oxidase(PC00175)	Qsox2
oxidoreductase(PC00176)	Me3
phosphodiesterase(PC00185)	Prune1 Pde10a
phospholipase(PC00186)	Pla2g4a
transferase(PC00220)	Hs6st1
**protein-binding activity modulator (PC00095)**	
G-protein(PC00020)	Arf2
GTPase-activating protein(PC00257)	Arhgap25 Sipa1l1
guanyl-nucleotide exchange factor(PC00113)	Trio Cyth1
heterotrimeric G-protein(PC00117)	Gng4
kinase activator(PC00138)	Ccnc
kinase modulator(PC00140)	Prkar1a
protease inhibitor(PC00191)	Serpinb6 Lxn
small GTPase(PC00208)	Rab27b Rhoq Rap2b Rab11fip5 Rab4a
**transporter (PC00227)**	
ATP synthase(PC00002)	Atp5f1d Atp5mk
ion channel(PC00133)	Cnnm2
primary active transporter(PC00068)	Mfsd12
transporter(PC00227)	Kpna1 Slco3a1 Slc16a3 Pitpnc1 Slc20a2 Tnpo1
voltage-gated ion channel(PC00241)	Cbarp Kcnn4 Vdac3
**cytoskeletal protein (PC00085)**	
actin binding motor protein(PC00040)	Myo5a Tpm4
actin or actin-binding cytoskeletal protein(PC00041)	Pdlim1 Arpc3
cytoskeletal protein(PC00085)	Tmem202 Whrn
microtubule binding motor protein(PC00156)	Dctn4
microtubule or microtubule-binding cytoskeletal protein(PC00157)	Wdr90

Continued on next page

Table S3 – continued from previous page

Protein Classes	Associated Genes
non-motor actin binding protein(PC00165)	Frmd5 Scin Coro1c
**transmembrane signal receptor (PC00197)**	
G-protein coupled receptor(PC00021)	Grm5 Gpr63 Lpar2 Mrgprx2 Gpr85 P2ry10
transmembrane signal receptor(PC00197)	Tlr4 Csf2rb2 Fzd6 Fzd7 Csf2rb
**scaffold_adaptor protein (PC00226)**	
scaffold/adaptor protein(PC00226)	Rassf3 Ywhaz Sntb2 Micall2 Klhl23 Mpp7 Mfhas1 Sh2b2 Plin2 Arrb1

Table S4: Summary of top two most abundant protein classes among downregulated ADO-specific genes.

Protein Classes	Associated Genes
**gene-specific transcriptional regulator (PC00264)**	
C2H2 zinc finger transcription factor(PC00248)	Glis2 Zfhx2 Zeb1 Zfp619 Zfp248 Ikzf2
DNA-binding transcription factor(PC00218)	Gata1 Nfix Gata2
HMG box transcription factor(PC00024)	Cic
basic helix-loop-helix transcription factor(PC00055)	Hes1
homeodomain transcription factor(PC00119)	Hoxb4
transcription cofactor(PC00217)	Crtc1 Arid4b
winged helix/forkhead transcription factor(PC00246)	Rfx5 Rfx7
**protein modifying enzyme (PC00260)**	
cysteine protease(PC00081)	Pgpep1 Uchl3 Usp20
non-receptor serine/threonine protein kinase(PC00167)	Ksr1 Taok3 Map4k2 Stk39 Prkcd Mink1
protein modifying enzyme(PC00260)	P4ha1
serine protease(PC00203)	Mbtps1
ubiquitin-protein ligase(PC00234)	Pias2 Trim58 Itch Rnf227

Table S5: Summary of genes involved in inflammatory mediator biosynthesis and their receptors in mast cells

Category	Associated Gene (Ligand)	Receptor(s)
Lipid Mediator Synthesis		
Precursor Liberation	Pla2g4a, Pla2g6	
2-arachidonoylglycerol (2-AG) synthesis	Dagla, Daglb	
Cyclooxygenase	Ptgs1, Ptgs2, Hpgds, Ptgds, Ptges, Ptges2, Ptges3, Tbxas1	
Lipoxygenase	Alox5, Alox5ap, Ltc4s, Lta4h, Alox12, Alox15	
Platelet-Activating Factor	Lpcat1, Lpcat2	
Cytochrome P450	Cyp2j5, Cyp2j6	
Cytokines, Chemokines, & Growth Factor		
Chemokine Superfamily	Ccl2	Ccr2
	Ccl3	Ccr1, Ccr5
	Ccl4	Ccr5
	Ccl5	Ccr1, Ccr3, Ccr5
	Ccl7	Ccr1, Ccr2, Ccr3
	Ccl8	Ccr2
	Ccl11	Ccr3
	Ccl12	Ccr2
	Ccl24	Ccr3
	Cxcl1	Cxcr2
	Cxcl2	Cxcr2
	Cxcl3	Cxcr2
	Cxcl5	Cxcr2
	Cxcl9	Cxcr3
	Cxcl10	Cxcr3
	Cxcl11	Cxcr3
	Cxcl12	Cxcr4
	Xcl1	Xcr1
	Cx3cl1	Cx3cr1
Interleukin (IL) Family	Il1a, Il1b, Il1f10	Il1r1/Il1rap
	Il2, Il4, Il7, Il9, Il15	Il2rg (common γ-chain)
	Il3	Il3ra/Csf2rb/Csf2rb2
	Il4, Il13	Il4ra/Il13ra1/Il2rg
	Il5	Il5ra/Csf2rb/Csf2rb2
	Il6	Il6r/Il6st
	Il9	Il9r/Il2rg

Continued on next page

Table S5 – *Continued from previous page*

Category	Associated Gene (Ligand)	Receptor(s)
	Il10	Il10ra/Il10rb
	Il12a, Il12b	Il12rb1/Il12rb2
	Il17a, Il17f	Il17ra/Il17rc
	Il18	Il18r1/Il18rap
	Il19, Il20, Il24	Il20ra/Il20rb
	Il22	Il22ra1/Il10rb
	Il23a, Il12b	Il23r/Il12rb1
	Il27	Il27ra/Il6st
	Il31	Il31ra
	Il33	Il1rl1
	Il34	Csf1r
TNF Superfamily	Tnf	Tnfrsf1a, Tnfrsf1b
	Lta	Tnfrsf1a
	Tnfsf4	Tnfrsf4
	Tnfsf9	Tnfrsf9
	Tnfsf10	Tnfrsf10b
	Fasl	Fas
	Cd40lg	Cd40
Interferon (IFN) Family	Ifna (all subtypes), Ifnb1	Ifnar1/Ifnar2 (Type I)
	Ifng	Ifngr1/Ifngr2 (Type II)
	Ifnl2, Ifnl3	Ifnlr1/Il10rb (Type III)
TGF-β Superfamily	Tgfb1, Tgfb2, Tgfb3	Tgfbr1/Tgfbr2
	Bmp2, Bmp4, Bmp7	Bmpr1a/Bmpr1b/Bmpr2/Acvr2a/Acvr2b
	Inhba, Inhbb	Acvr1b/Acvr2a/Acvr2b
	Tgfa	Egfr
CSFs	Csf1	Csf1r
	Csf2	Csf2ra/Csf2rb/Csf2rb2
	Csf3	Csf3r
Wnt Family	Wnt1, Wnt2, Wnt2b, Wnt3, Wnt3a, Wnt4, Wnt5a, Wnt5b, Wnt6, Wnt7a, Wnt7b, Wnt8a, Wnt8b, Wnt9a, Wnt9b, Wnt10a, Wnt10b, Wnt11, Wnt16	Fzd1-10, Lrp5, Lrp6
Other Growth Factors	Vegfa	Kdr, Flt1
	Vegfb	Flt1
	Pdgfa, Pdgfb	Pdgfra/Pdgfrb
	Edn1	Ednra
	Flt3l	Flt3
	Kitl	Kit

Continued on next page

Table S5 – *Continued from previous page*

**Category**	**Associated Gene (Ligand)**	**Receptor(s)**
	Lif	Lifr
	Osm	Osmr
	Thpo	Mpl
	Tslp	Crlf2/Il7r
Other Secreted Proteins	Hmgb1	Ager, Tlr2, Tlr4
	Mif	Cd74
